# The Effect of *Ustilago maydis* and Delayed Harvesting on A- and B-Type Trichothecene Concentrations in Maize Grain

**DOI:** 10.3390/jof9080794

**Published:** 2023-07-28

**Authors:** Eimantas Venslovas, Audronė Mankevičienė, Yuliia Kochiieru, Sigita Janavičienė, Zenonas Dabkevičius, Vadims Bartkevičs, Zane Bērziņa, Romans Pavlenko

**Affiliations:** 1 Lithuanian Research Centre for Agriculture and Forestry, Instituto al. 1, LT-58344 Akademija, Lithuania; 2 Institute of Food Safety, Animal Health and Environment “BIOR”, Lejupes Iela 3, LV-1076 Riga, Latvia

**Keywords:** common smut, *Fusarium* spp., field experiment, maize hybrids, deoxynivalenol, 3-acetyl-deoxynivalenol, 15-acetyl-deoxynivalenol, T-2 toxin, HT-2 toxin

## Abstract

The aim of this study was to investigate whether, in the context of a higher incidence of *Ustilago maydis* and *Fusarium* spp. at optimal and delayed harvest times, a higher incidence of mycotoxin contamination in maize grains could be expected. The field experiment was carried out at the Lithuanian Research Centre for Agriculture and Forestry experimental fields over three consecutive years (2020–2022). Two maize hybrids (*Duxxbury* and *Lapriora*) with different FAO numbers were used. The experimental design in the field was a randomized complete block design. Harvesting took place at three different times: first at physiological maturity, and then 10 (±2) and 20 (±2) days after the first harvest. Each hybrid had four repetitions at different harvest times. The *U. maydis* infection was only detected in 2021 and after the first harvest cobs were further divided into four different groups with four repetitions: healthy cobs, cobs visually infected with Fusarium spp., cobs visually infected with common smut, and cobs visually infected with both pathogens. No *U.* maydis-damaged maize cobs were found in 2020 and 2022. The levels of *Fusarium* microscopic fungi in maize grains were also from 4 to 16 times higher in 2021 than in 2020 and 2022. Harvest delays in 2020 led to a significant deoxynivalenol concentration increase in the *Duxxbury* hybrid and an HT-2 concentration increase in the *Lapriora* hybrid. In 2021, deoxynivalenol, 3-acetyl-deoxynivalenol, 15-acetyl-deoxynivalenol, and HT-2 concentrations significantly rose in both hybrids, but the T-2 concentration significantly increased only in the *Lapriora* hybrid. Deoxynivalenol concentrations were, respectively, 110 and 14.6 times higher than in cobs only infected with *Fusarium* spp. or *U. maydis*. Concentrations of 15-acetyl-deoxynivalenol were, respectively, 60, 67, and 43 times higher than in asymptomatic cobs and cobs only infected with *Fusarium* spp. or *U. maydis*. Cobs contaminated with both pathogens also had higher concentrations of 3-acetyl-deoxynivalenol. T-2 and HT-2 were detected in maize grains harvested from cobs infected only with *Fusarium* spp. The presence of *U. maydis* and *Fusarium* fungi in maize cobs, along with harvest delays, led to significant increases in mycotoxin concentrations, highlighting the importance of timely harvesting and pathogen management to mitigate mycotoxin contamination in maize grains.

## 1. Introduction

Maize (*Zea mays L.*) is one of the most adaptable crops, able to thrive in a variety of environments, and used for human food and animal feed [1]. According to recent research, maize is the world’s second most extensively farmed crop after wheat [2]. Globally, it is expected for grain production to increase over the next decade by 12%, and almost half of this increase will come from maize. The global grain consumption for feed is also expected to be dominated by maize [3]. Maize yields in Europe, and especially in northern Europe, are expected to grow even further, despite climate change [4]. Over the last decade, maize yields in Lithuania have already gone up from 78.8 to 99.8 thousand tons [5].

Common smut is caused by the basidiomycete fungus, *Ustilago maydis* [6]. It is a common biotrophic phytopathogenic fungus, which specifically infects maize organs by forming galls filled with teliospores [7]. Unfavorable meteorological conditions, such as high temperatures and droughts in the period of pollen scattering and filament spreading, have an impact on the spread of common smut. The disease is also more intense when young tissue is damaged by mechanical damage, wind, or hail [8,9]. This disease can impede plant development and diminish production, resulting in economic losses of up to 10% [6,7].

Considering the varying degrees of resistance exhibited among different maize hybrids, it is recommended to prioritize the selection of less susceptible hybrids to effectively protect the crop [10]. Resistance to *U. maydis* is believed to be a quantitative trait influenced by multiple minor gene effects. However, the specific genes and intricate mechanisms underlying maize resistance to *U. maydis* remain largely uncharacterized [11]. Hence, considerable efforts are being devoted to the development of novel hybrids that exhibit enhanced resilience, while also recognizing the importance of identifying existing hybrids that already demonstrate higher levels of resistance [12].

*U. maydis* does not produce dangerous metabolites; however, smut galls on maize cobs rupture the husks and offer a path for other fungi to infect exposed, unsmutted kernels [9,13]. More recent studies have reported that maize smut galls can also be colonized by mycotoxigenic fungi and contaminated with mycotoxins [14]. Other researchers claim that mycotoxins can also be detected in varying amounts in canned smut galls [15]. However, in some countries, such as Mexico, it is considered a delicacy and an important protein (~12%) source that has several names: “maize mushroom”, “corn truffle”, “cuitlachoche”, and “huitlacoche” [16,17].

One of the most important mycotoxin producers is *Fusarium* fungi. Species such as *F. graminearum* and *F. culmorum* mainly produce type B trichothecenes deoxynivalenol (DON), 3-acetyl-deoxynivalenol (3ADON), and 15-acetyl-deoxynivalenol (15ADON). Species such as *F. langsethiae*, *F. poae*, *F. equiseti*, and *F. sporotrichioides* mainly produce type A trichothecenes T-2, HT-2 [18,19]. Research shows that DON can be detected in almost half of the samples tested, and maize is among the crops with the highest concentrations of DON. More data should also be collected on 3ADON and 15ADON to better characterize their potential contribution to the overall impact of DON [20]. Further collection of analytical data on T-2 and HT-2 in relevant food and feed commodities, with particular focus on analyzing both individual toxins in the same sample, is also encouraged [21].

Grain can be influenced by unfavorable environmental conditions (temperature, humidity, drought, and rainfall) at any stage of the production process: pre-harvest, at harvest, and during storage [22,23]. Higher precipitation and lower temperatures before harvest and the delayed harvest of maize in different climate zones than ours have been observed to result in higher levels of Fusarium spp. and elevated mycotoxin contamination [24,25,26]. However, there is still a lack of information about the impact of delayed harvest time on maize grain infestation in our region.

The consumption of grains that are contaminated with high levels of mycotoxins can cause chronic, acute illness or even death in both humans and animals [27]. The European Commission has therefore set maximum limits for certain mycotoxins in food and feed [28,29,30]. As heavily infested grains are unsuitable for consumption and the detoxification/decontamination of such grains is a global problem, both practically and scientifically, it can also lead to serious economic losses [31]. It is therefore very important to continuously monitor, assess, and avoid conditions that may increase mycotoxin concentrations [19].

The fast expansion of maize farming areas, the use of ineffective crop rotation, and the global warming environment have all contributed to a rise in the occurrence of *Fusarium* spp. and common smut (*U. maydis*) [32]. Therefore, the aim of this study was to investigate whether, in the context of a higher incidence of common smut and *Fusarium* spp. at optimal and delayed harvest times, a higher incidence of mycotoxin contamination in maize grains is expected.

## 2. Materials and Methods

### 2.1. Field Trial Experimental Design

The research was carried out in the experimental fields of the Lithuanian Research Centre for Agriculture and Forestry between 2020 and 2022. The experimental design in the field was a randomized complete block design. Two corn hybrids, *Lapriora* (FAO 190) and *Duxxbury* (FAO 170), for three different planned harvest times were sown. Each treatment was replicated in four blocks (twenty-four blocks in total). To control weeds, maize was sprayed with herbicide ESTET^®^ 600 EC (active substance 2.4 D acid 600 g L^−1^) 0.6 L ha^−1^ one month after the seeding and repeatedly after two weeks with Nicogan^®^ (active substance nicosulfuron 40g L^−1^) 0.75 L ha^−1^ (BBCH 13-19). No other plant protection products were used. The first harvest was carried out when the maize reached physiological maturity (BBCH 87). The second maize harvest took place 10 (±2) days and the third harvest took place 20 (±2) days after the first harvest. The corn cobs were harvested and shelled by hand. Grains were dried to a moisture content of around 13%, and some grains were milled using Ultra Centrifugal Mill ZM 200 (Retsch, Haan, Germany) with 0.8 mm sieve. The milled and whole grains were frozen in a freezer at −20 °C until further laboratory analyses.

### 2.2. Meteorology

**Figure 1:**
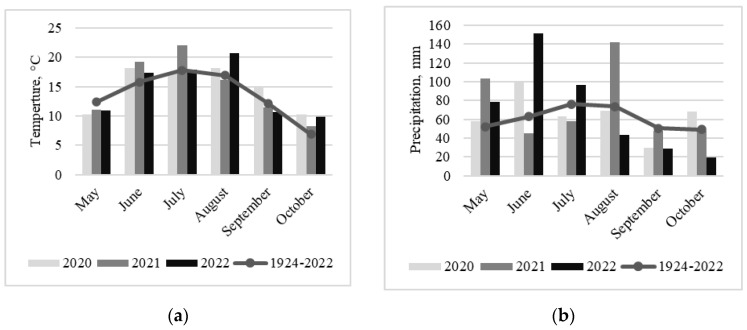
The average monthly air temperature (**a**) and precipitation (**b**) during the 2020–2022 maize growing seasons (May–October) and long-term average (1924–2022).

In 2020, temperatures at the beginning of the maize growing season in June were above the long-term average (Figure 1a). However, temperatures were cooler during flowering compared to the other years in the study. The late summer and early autumn were warmer than usual. Precipitation at the beginning of the maize growing season was higher; however, during flowering, late summer and autumn rainfalls were lower than the long-term average (Figure 1b). The year of 2021 was exceptional, with temperatures well above the long-term average and very low precipitation at the beginning of the summer, as well as during flowering and silking of maize. The end of the growing season was much cooler and wetter compared to the 2020 and 2022 meteorological data. In 2022, the temperature at the beginning of the maize growing season was close to the long-term average, and the precipitation was higher compared to the other years in the study. The end of the summer was exceptionally warm and dry, and early autumn was also drier than usual.

### 2.3. Ustilago Maydis Rating

Infection with *U. maydis* was assessed before the first harvest. *U. maydis* was evaluated by counting diseased and healthy maize cobs on five 2 m lengths of the row selected randomly for each hybrid [33]. During the whole study period, *U. maydis* infection was only noticed in 2021, and in turn, in 2020 and 2022, no maize cobs were infected. Therefore, in 2021, the first harvest cobs were further divided into four different groups: healthy cobs, cobs visually infected with *Fusarium* spp., cobs visually infected with common smut, and cobs visually infected with both pathogens. Cobs visually infected with common smut had tumor-like galls, and cobs infected with *Fusarium* spp. had white to pink, salmon-colored, cottony mold on multiple grains. Each group had four replicates; each replicate was prepared in two repetitions. Grains were dried and milled, and mycotoxin analyses were carried out.

### 2.4. Fusarium spp. Rating

An agar plate method was used for the estimation of internal grain infection. The grain surface was sterilized for 3 min in 1% NaOCl solution, then 100 grains per sample were plated in Petri dishes with a potato dextrose agar (PDA) and incubated for 7 days at 26 ± 2 °C in the dark [34]. The overgrown *Fusarium* colonies were isolated and purified. To identify the colonies, the manuals of Nelson et al. [35] and Leslie et al. [36] were used. An optical microscope Nikon Eclipse E200 (Nikon, Tokyo, Japan) was used to identify the *Fusarium* spp. fungus, and the contaminated grains were calculated in percent (0% represents all healthy grains; 100% represents all infected grains).

### 2.5. Mycotoxin Analyses

In 2020 and 2021, for the sample clean-up step and dilution (in case of high concentrations), Vicam DONtest^TM^ WB and T-2/HT-2^TM^ LC immunoaffinity columns (Milford, MA, USA) were used, according to the manufacturer’s procedures. DON test antibodies cross-react with 3ADON and 15ADON and succeed in retaining DON and the two other conjugates with good recoveries [37]. Therefore, the DONtest^TM^ WB columns were also used to determine 3ADON and 15ADON.

The mycotoxin analyses were carried out using Shimadzu (Kyoto, Japan) high-performance liquid chromatography (HPLC) system. The system consists of an autosampler SIL-20A, a degasser DCU-20A5, a LC20 AT pump equipped with a FCV-10AL quaternary valve, a degasser DCU-20A5, a column oven CTO-20A equipped with a YMC-Pack Pro C18, (150 mm × 4.0 mm, 3 µm) column, an FLD detector, and a UV detector (Shimadzu). Data were evaluated using the computer program LCsolution LC/GC, version 5.42 (Shimadzu).

The quantitation of DON, 3ADON, and 15ADON in the sample was performed by measuring the peak area at DON and its derivates’ retention time and comparing it with the standard curves. For DON and 3ADON, calibration curve standard solutions were prepared with concentrations of 0.1, 0.2, 0.5, 1, 2, 3.125, and 5 µg mL^−1^, for 15ADON, concentrations of 0.1, 1, 3.4, 5, 10, 34, and 50 µg mL^−1^, and for T-2 and HT-2, concentrations of 0.01, 0.05, 0.1, 0.25, and 0.5 µg mL^−1^. Coefficient of determination (r^2^) was not less than 0.999 in all cases. The lower limit of detection (LOD) and limit of quantification (LOQ) in ng g^−1^ were calculated for DON (LOD = 37, LOQ = 112), 3ADON (LOD = 19, LOQ = 64), 15ADON (LOD = 19, LOQ = 63), T-2 (LOD = 15, LOQ = 50), and HT-2 (LOD = 19, LOQ = 62).

Grain samples in 2022 were analyzed using the instrumental method based on HPLC coupled to tandem quadrupole mass spectrometry (MS/MS) [38]. The mycotoxin analyses in 2022 were carried out using UltiMate 3000 HPLC system (Waltham, MA, USA) coupled with Thermo TSQ Quantiva triple quadrupole mass spectrometer (Waltham, MA, USA). Positive and negative ion modes were used to monitor ions, and selected reaction monitoring mode was used for the mass analysis. Xcalibur^TM^ and TraceFinder software were used to process the data.

For DON, calibration curve standards with concentrations of 10, 50, 100, 250, and 500 ng g^−1^, and for 3ADON, 15ADON, T-2, and HT-2, concentrations of 10, 20, 50, 100, and 200 ng g^−1^ were used. The coefficient of determination (r^2^) was not less than 0.999 in all cases. The LOD and LOQ in ng g^−1^ were calculated for DON (LOD = 48; LOQ = 144); sum of 3ADON and 15ADON (LOD = 17; LOQ = 51), T-2 (LOD = 17; LOQ = 52), and HT-2 (LOD = 10; LOQ = 31).

The performance characteristics of both analytical procedures in terms of sensitivity, precision, and accuracy were similar, as the presented data reveals that methods offer very similar LOD and LOQ values.

### 2.6. Statistical Analysis

Statistical analysis was conducted using SPSS Statistics, version 25 (IBM Inc., Armonk, NY, USA). To assess the assumptions of homoscedasticity, Levene’s test was applied, and data normality was checked using the Shapiro–Wilk test. These tests confirmed that the data met the assumptions required for conducting ANOVA, ensuring the validity and reliability of our statistical analysis. Significant differences of *Fusarium* spp. infection and mycotoxin concentrations between treatments were calculated using one-way ANOVA (Duncan’s post hoc test). Significant differences of common smut infection between maize hybrids were calculated using T-test. Pearson’s correlation coefficient was used to determine positive and negative correlations and their significance between *Fusarium* species and mycotoxins.

## 3. Results

Common smut and *Fusarium* infection were observed in maize cobs and grains in Lithuania in each of the years from 2020 to 2022. The study showed that in 2021, the meteorological conditions were much more favorable for the spread of both *Fusarium* fungi and *U. maydis*.

**Figure 2:**
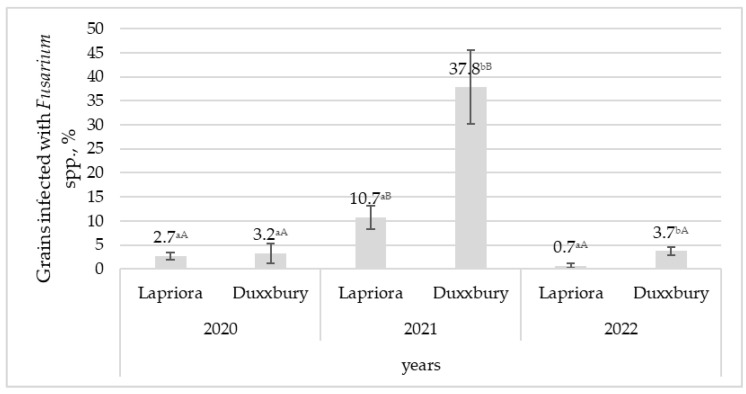
Percentage of grains infected with *Fusarium* spp. in *Lapriora* and *Duxxbury* maize hybrids over a three-year period (2020–2022). Note. Values with different lowercase and uppercase letters indicate significant differences among maize hybrids in different years for the same infection.

The *U. maydis* infection was only detected in 2021, and no damaged maize cobs were found in 2020 and 2022 (*p* < 0.001). Differences between maize hybrids were observed in the year of disease incidence, and the *Duxxbury* hybrid had almost four times more smut-infected cobs than the *Lapriora* hybrid (*p* < 0.001). In 2021, the levels of *Fusarium* microscopic fungi in both hybrids were from 4 to 16 times higher than in 2020 and 2022 (*p* < 0.001) (Figure 2). In 2021 and 2022, it was observed that *Fusarium* microscopic fungi were approximately four times more abundant in the *Duxxbury* hybrid than in the *Lapriora* hybrid (*p* < 0.01); however, in 2020, no significant differences were observed between the hybrids.

**Figure 3:**
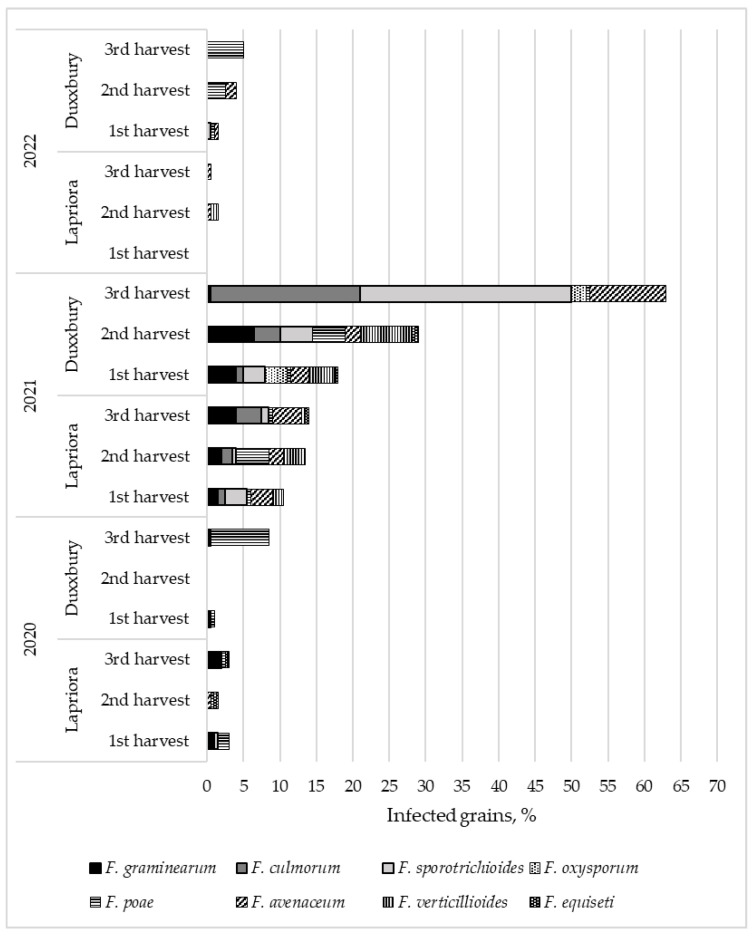
Variation in percentage of grains infected with different *Fusarium* species in two maize hybrids (*Lapriora* and *Duxxbury)* harvested at physiological maturity, then 10 (±2) and 20 (±2) days after the first harvest over a three-year period (2020–2022).

While examining the species composition of *Fusarium* fungi in maize hybrids at different harvest times, we observed differences in the diversity of microscopic fungi detected in maize grain (Figure 3). The lowest diversity of *Fusarium* fungi was found in the grains of the *Duxxbury* hybrid in 2020 and in the grains of both hybrids in 2022. The highest diversity was observed in the *Lapriora* hybrid in 2020 and both hybrids in 2021. In 2021, the number of infected grains in both hybrids was much higher compared to the results of the other two years. Duxbury hybrid grains in 2021 were 12 and 10 times more infected than in 2020 and 2022, respectively, and *Lapriora* hybrid grains were 4 and 16 times more infected than in 2020 and 2022, respectively (*p* < 0.001). The microscopic fungi that dominated were *F. graminearum*, *F. culmorum*, *F. sporotrichioides*, *F. avenaceum*, and *F. verticillioides*. A statistically significant increase in total microscopic fungi was only observed in the third harvest of *Duxxbury* in 2021, when the amount of infected grain was found to be three times higher than in the first harvest and two times higher than in the second harvest (*p* < 0.01).

In 2020 and 2021, DON, T-2, and HT-2 toxins were detected in all maize grain samples, whereas in 2022, traces of DON and HT-2 toxins were detected in only 21% and 8% of samples (Table 1). 3ADON and 15ADON were not detected in 2020 and 2022; however, in 2021, 3ADON was found in 46% of grain samples and 15ADON was found in 75% of grain samples. Comparing the mycotoxin concentrations detected in maize grain between the years, it was observed that DON, 3ADON, and 15ADON concentrations in both hybrids (*p* < 0.05) and T-2 and HT-2 concentrations only in *Duxxbury* hybrid (*p* < 0.001) were significantly higher in 2021 than in 2020 and 2022. In the same year, differences between the hybrids also became apparent. It was observed that the concentrations of DON, 3ADON, T-2, and HT-2 were 5 (*p* < 0.01), 6 (*p* < 0.05), 4 (*p* < 0.05), 26 (*p* < 0.05), and 15 (*p* < 0.001) times higher in the *Duxxbury* hybrid than in the *Lapriora* hybrid, respectively. With delayed harvesting, the concentrations were observed to increase or remain stable. Significant increases in concentrations are usually observed at the third harvest. In 2020, harvest delay led to a significant DON concentration increase in the *Duxxbury* hybrid and a HT-2 concentration increase in the *Lapriora* hybrid (*p* < 0.05). In 2021, a significant concentration-increasing tendency in delayed harvesting was observed in DON, 3ADON, 15ADON, and HT-2 concentrations in both hybrids (*p* < 0.05), while the T-2 concentration significantly increased only in the *Lapriora* hybrid (*p* < 0.05). In 2021, the DON concentration in 42% of samples and the T-2 and HT-2 concentration sums in 50% of samples were above the levels set by the European Commission for animal feed.

Several positive correlations were observed between type A and B trichothecenes and *Fusarium* spp. fungi (Table 2). The levels of DON, 3ADON, and 15ADON indicated the strongest positive correlations with *F. culmorum* and *F. sporotrichioides* (*p* < 0.001). The content of T-2 weakly but statistically significantly positively correlated with *F. graminearum* and *F. sporotrichioides* (*p* < 0.05). For HT-2, the strongest correlation was noticed with *F. sporotrichioides* (*p* < 0.001); it also strongly correlated with *F. graminearum* and *F. culmorum* (*p* < 0.01).

By analyzing cobs collected in 2021 and visually distributed by asymptomatic, infected with *U. maydis*, infected with *Fusarium* spp., and infected with both pathogens, it was determined that grains harvested from cobs infected with both had the highest DON, 3ADON, and 15ADON concentrations (Table 3). DON concentrations were, respectively, 110 and 14.6 times higher than in cobs that were only infected with *Fusarium* spp. or *U. maydis* (*p* < 0.001). 15ADON concentrations were, respectively, 60, 67, and 43 times higher than in asymptomatic cobs and cobs only infected with *Fusarium* spp. or *U. maydis* (*p* < 0.001). High concentrations of 3ADON were also detected in cobs contaminated with both pathogens, and some only *U. maydis*-infected cob samples had traces of up to 28 ng g^−1^. Unlike the mycotoxins discussed above, higher concentrations of T-2 and HT-2 were detected in maize grains harvested from cobs infected only with *Fusarium* spp. However, concentrations of T-2 and HT-2 up to 23 and 24 ng g^−1^ were detected in grains harvested from cobs contaminated only with *U. maydis*. Moreover, 50% of the grain samples harvested from cobs infected with both pathogens also had T-2 and HT-2 concentrations of up to 24 and 57 ng g^−1^, respectively.

## 4. Discussion

In recent years, there has been an upward trend in the temperature background, sharp fluctuations in humidity, and the occurrence of extreme weather events. This stresses the plants and makes them less resistant to pests [39]. Any mechanical plant damage from pests, wind, or hail leads to a higher infestation with pathogens, and maize cobs are often infected when the spores of any pathogen are dispersed by wind onto the cob silks [9]. The most recent research, conducted in Poland and Ukraine, has also shown that warmer and drier summers can lead to a higher incidence of *Fusarium* diseases and common smut [8,39]. Therefore, in 2021, dry, hot, and windy weather during the flowering and silking of maize may have led to higher infestations of *Fusarium* fungi and common smut.

Several methods can be employed to protect maize from pathogens. These include implementing crop rotation and tillage practices to reduce inoculum from plant debris, ensuring an appropriate planting population, maintaining optimal watering from silk to late-dough stage, and achieving optimal nitrogen fertilization [9]. Nevertheless, the degree of infection is also influenced by hybrid resistance to specific pathogens. The research conducted in Hungary indicated that common smut infection exhibits divergent effects on various maize hybrids, particularly emphasizing the higher impact on sweet maize hybrids [10]. Therefore, developing hybrids that are resistant to pathogens through breeding or genetic engineering is crucial to establishing a foundation for sustainable agriculture [12].

The findings from researchers in China suggest that the resistance to maize common smut may be attributed to intricate gene co-expression and metabolism networks associated with amino acids and reactive oxygen species metabolism [10]. German research demonstrates that breeding or engineering maize hybrids with enhanced resistance, specifically targeting the susceptibility factor lipoxygenase 3 through gene-editing techniques, holds promise for reducing disease symptoms and fungal infections caused by *U. maydis* [12]. The hybrids included in our study had not undergone previous testing for resistance to *U. maydis*. However, our findings indicate that the *Lapriora* hybrid exhibited greater resistance to this pathogen under the same growth conditions, highlighting its potential as a valuable candidate for further investigation and utilization in disease management strategies.

Maize grains can be infected with various species of *Fusarium* fungi, such as *F. graminearum*, *F. culmorum*, *F. langsethiae*, *F. poae*, *F. equiseti*, *F. sporotrichioides*, and *F. verticillioides* [18,19]. The studies conducted in Poland confirmed that *F. verticillioides*, *F. culmorum*, *F. graminearum*, *F. sporotrichioides*, and *F. poae* are among the most common *Fusarium* species in maize [40,41].

There is still a lack of information about the impact of a delayed harvest time on maize grain infestation in our region for mycotoxins. However, in sub-tropical/tropical Brazil and Uganda climate zones, it was noticed that delays in maize harvest can increase disease severity and *Fusarium* spp. and mycotoxins, such as total aflatoxin and fumonisin, contamination [24,25]. Research in Italy showed that higher precipitation and lower temperatures can lead to higher *Fusarium* incidence and mycotoxins such as DON, aflatoxin B1, fumonisin B1 + B2, and zearalenone [26]. A study in Serbia also showed that a wet and rainy climate in one of the study years led to an increase in the concentrations of DON and its derivatives [22]. Therefore, in 2021, more rainy and cooler weather in maize pre-harvest and harvest periods might have led to higher contamination of already heavily infested grain. As trichothecenes of types A and B are mainly produced by *F. graminearum*, *F. culmorum*, *F. langsethiae*, *F. poae*, and *F. sporotrichioides* [18,19]. Other researchers have also noticed a positive correlation between *F. culmorum*, *F. graminearum* occurrence, and type A and B trichothecenes concentrations and between *F. sporotrichioides* occurrence and T-2 and HT-2 concentrations in maize and other cereals [42,43,44].

There are still no studies on whether *Fusarium* fungi together with common smut infecting the same cob can lead to higher levels of trichothecenes in maize grain. However, research conducted in the USA has shown that grains harvested from common smut-infected cobs have 45 times higher aflatoxin and more than 5 times higher fumonisin concentrations than smut-free cobs [13]. As common smut does not produce mycotoxins, researchers found that *Aspergillus* spp. could grow on dried common smut galls, and there is a potential for aflatoxin contamination [31]. Mycotoxins such as aflatoxin, fumonisin, and DON can be found in commercially canned and fresh common smut galls [15]. Moreover, a loss of husk integrity caused by *U. maydis* makes the adjacent asymptomatic grains susceptible to attack by other fungi [13]. This may have led to a higher increase in the concentrations of some trichothecenes in maize grains harvested from cobs infected by both *Fusarium* fungi and common smut.

## 5. Conclusions

This study reveals that the presence of Ustilago maydis and Fusarium fungi in maize cobs is associated with significant increases in mycotoxin concentrations in maize grains. Additionally, harvest time delays further exacerbate mycotoxin contamination. Dry and warm weather during the flowering and silking seasons of maize can lead to a significantly higher infestation rate with *Fusarium* fungi and common smut. If the environmental conditions are favorable for the infection of maize cobs, the delay in harvesting during rainy and cooler weather may increase the infestation and the concentrations of DON, 3ADON, 15ADON, T-2, and HT-2 mycotoxins. The infection of cobs with common smut may lead to easier infection with other mycotoxigenic fungi and a significant increase in DON, 3ADON, and 15ADON concentrations in maize grains harvested from such cobs. These findings underscore the critical importance of timely harvesting and implementing effective pathogen management strategies to mitigate mycotoxin contamination in maize production. Given the diversity and quantity of the mycotoxins detected in this study, it is appropriate to carry out a more extensive survey of maize crops where common smut and *Fusarium* fungi predominate.

## Figures and Tables

**Table 1:** A- and B-type trichothecenes concentration in *Lapriora* and *Duxxbury* maize hybrids harvested at physiological maturity, then 10 (±2) and 20 (±2) days after the first harvest over a three-year period (2020–2022).

Year	*Lapriora* Hybrid			*Duxxbury* Hybrid		
	First Harvest	Second Harvest	Third Harvest	First Harvest	Second Harvest	Third Harvest
	DON					
2020	198 ^a^	229.3 ^a^	272.5 ^a^	149.8 ^a^	202.3 ^ab^	232.5 ^b^
2021	227 ^a^	518 ^a^	3481 ^b^	1880 ^a^	4456 ^a^	15019 ^b^
2022	13 ^a^	<LOD	69 ^a^	346 ^a^	192 ^a^	<LOD
	3ADON					
2020	<LOD	<LOD	<LOD	<LOD	<LOD	<LOD
2021	<LOD	<LOD	64	31 ^a^	40 ^a^	322 ^b^
	15ADON					
2020	<LOD	<LOD	<LOD	<LOD	<LOD	<LOD
2021	<LOD	35 ^a^	246 ^b^	109 ^a^	169 ^a^	968 ^b^
	Sum of 3ADON and 15ADON					
2022	<LOD	<LOD	<LOD	<LOD	<LOD	<LOD
	HT-2					
2020	49.9 ^a^	34.8 ^a^	101.9 ^b^	50.2 ^a^	52.6 ^a^	41.2 ^a^
2021	22.1 ^a^	16.6 ^a^	154.2 ^b^	884 ^a^	830.9 ^a^	1196.8 ^b^
2022	<LOD	<LOD	<LOD	<LOD	<LOD	<LOD
	T-2					
2020	27.3 ^a^	27.8 ^a^	62.5 ^a^	23.2 ^a^	36.1 ^a^	23.6 ^a^
2021	24.6 ^a^	16.3 ^a^	174.4 ^b^	1960.1 ^a^	2098.5 ^a^	1413.2 ^a^
2022	<LOD	<LOD	<LOD	<LOD	<LOD	<LOD

Mycotoxin concentrations with different lowercase letters indicate significant differences among harvest times in the same maize hybrid and year.

**Table 2:** A- and B-type trichothecenes correlation with *Fusarium* spp. species in maize grains.

	DON	3ADON	15ADON	T-2	HT-2
*F. graminearum*	0.192	0.07	0.110	0.290 *	0.339 **
*F. culmorum*	0.731 ***	0.809 **	0.831 **	0.161	0.365 **
*F. sporotrichioides*	0.559 ***	0.333 **	0.358 **	0.272 *	0.445 ***
*F. poae*	−0.063	−0.064	−0.064	−0.031	0.026

*—*p* < 0.05, **—*p* < 0.01, ***—*p* < 0.001.

**Table 3:** A- and B-type trichothecenes concentration in maize grains harvested from asymptomatic cobs and from visually infected with *U. maydis*, *Fusarium* spp., or both pathogens.

	Infection	N	Positive Samples, %	Mean	SE	95% Confidence Interval for Mean		Min.	Max.
						Lower Bound	Upper Bound		
DON	Asymptomatic	8	0%	<LOD	NA	NA	NA	<LOD	<LOD
	*Ustilago maydis*	8	100%	285 ^b^	73.3	128.6	440.9	92	896
	*Fusarium* spp.	8	50%	<LOD	NA	NA	NA	<LOD	88
	Both pathogens	8	100%	4175 ^c^	32.4	4097.8	4251.2	4060	4280
3ADON	Asymptomatic	8	0%	<LOD	NA	NA	NA	<LOD	<LOD
	*Ustilago maydis*	8	25%	<LOD	NA	NA	NA	<LOD	28
	*Fusarium* spp.	8	0%	<LOD	NA	NA	NA	<LOD	<LOD
	Both pathogens	8	100%	993	124.6	697.8	1287.2	652	1344
15ADON	Asymptomatic	8	100%	49 ^a^	6.4	34.0	64.0	20	76
	*Ustilago maydis*	8	50%	44 ^a^	11.6	19.4	68.6	<LOD	120
	*Fusarium* spp.	8	100%	68 ^a^	8.5	47.9	88.1	40	96
	Both pathogens	8	100%	2929 ^b^	381.3	2027.3	3830.7	1872	4028
T-2	Asymptomatic	8	0%	<LOD	NA	NA	NA	<LOD	<LOD
	*Ustilago maydis*	8	25%	<LOD	NA	NA	NA	<LOD	23
	*Fusarium* spp.	8	50%	132	47.0	21.0	243.2	<LOD	259
	Both pathogens	8	50%	<LOD	2.9	6.7	20.3	<LOD	24
HT-2	Asymptomatic	8	0%	<LOD	NA	NA	NA	<LOD	<LOD
	*Ustilago maydis*	8	19%	<LOD	NA	NA	NA	<LOD	24
	*Fusarium* spp.	8	50%	72 ^b^	25.2	12.6	131.9	<LOD	143
	Both pathogens	8	50%	31 ^a^	9.4	8.5	52.7	<LOD	57

LOD—limit of detection; NA—not available; mycotoxin concentrations, with different lowercase letters indicate significant differences among groups.

## Data Availability

Not applicable.
